# Female-pattern hair loss: therapeutic update^⋆^

**DOI:** 10.1016/j.abd.2022.09.006

**Published:** 2023-03-30

**Authors:** Paulo Müller Ramos, Daniel Fernandes Melo, Henrique Radwanski, Rita Fernanda Cortez de Almeida, Hélio Amante Miot

**Affiliations:** a Department of Dermatology, Faculty of Medicine, Universidade Estadual Paulista, Botucatu, SP, Brazil; b Department of Dermatology, Universidade do Estado do Rio de Janeiro, Rio de Janeiro, RJ, Brazil; c Private clinic, Rio de Janeiro, RJ, Brazil

**Keywords:** Androgenetic alopecia, Female-pattern alopecia, Finasteride, Minoxidil, Treatment, Female pattern hair loss

## Abstract

Female androgenetic alopecia or female-pattern hair loss (FPHL) is highly prevalent and has a great impact on the quality of life. The treatment is a routine challenge in dermatological practice, as many therapeutic options have a limited level of evidence and often do not meet patients expectations. Lack of knowledge of the pathogenesis of the hair miniaturization process and the factors that regulate follicular morphogenesis restricts the prospect of innovative therapies. There is also a lack of randomized, controlled studies with longitudinal follow-up, using objective outcomes and exploring the performance of the available treatments and their combinations. Topical minoxidil, which has been used to treat female pattern hair loss since the 1990s, is the only medication that has a high level of evidence and remains the first choice. However, about 40% of patients do not show improvement with this treatment. In this article, the authors critically discuss the main clinical and surgical therapeutic alternatives for FPHL, as well as present camouflage methods that can be used in more extensive or unresponsive cases.

## Introduction

Female-pattern hair loss (FPHL), or female androgenetic alopecia (AGA), is characterized by a progressive miniaturization of hair follicles and decreased hair density, primarily in the central-parietal region of the scalp.^1^ A recent epidemiological study carried out in a Brazilian population showed an overall prevalence of FPHL of 32.3% (95% CI 27.4%–36.9%) among adult women, increasing with age: 8% (20‒29 years) to 68 % (60‒75 years). The severity of FPHL has been associated with sedentary lifestyle, systemic arterial hypertension, and living in an urban area.^2^

The pathogenesis of FPHL has not yet been fully elucidated, nor are the factors that modulate miniaturized follicle morphogenesis known.^1^ In addition to the role of androgens, most clearly demonstrated in male AGA, there is evidence of the participation of genetic, hormonal, and environmental elements.^1^, ^3^, ^4^

For women, dense and healthy hair implies feelings of self-esteem, self-confidence, reflects their ability to change, security, and implies social interaction. These elements, associated with disease chronicity, and its therapeutic refractoriness, have an important negative impact on womens quality of life.^5^

Even though several treatments have already been proposed for FPHL, in a recent meta-analysis, only topical minoxidil accumulated an adequate level of evidence.^6^ In fact, there is scarcity of randomized, controlled studies with longitudinal follow-up and the use of objective outcomes that explore the performance of available treatments and their combinations.

Comparison between studies is also hindered by the use of different outcomes, time of follow-up, and the inclusion of different degrees of FPHL severity. Moreover, the direct inference of the results of clinical trials of male AGA for FPHL is not adequate.

The aim of this study is to describe and discuss the main therapeutic alternatives for FPHL, as well as to describe camouflage methods that can be used in more extensive or unresponsive cases.

## Clinical treatments

### Topical minoxidil

Minoxidil is a vasodilator initially used orally to treat high blood pressure. It was approved by the US Food and Drug Administration (FDA) for this purpose in 1979. Minoxidil activates the ATP-sensitive potassium channel (K^+^ ATP channel) causing hyperpolarization, efflux of K^++^ ions, and vessel smooth muscle relaxation.^7^

When used to treat high blood pressure, approximately 80% of the patients develop hypertrichosis. It was described as effective in improving male AGA in 1980.^8^ After the first observations, minoxidil started to be studied as a topical solution for the treatment of AGA, initially in men and later in women.

The mechanism of action of minoxidil in the treatment of AGA has yet to be fully elucidated.^9^ In addition to its vasodilator action, minoxidil increases proliferation in cultured cells of the dermal papilla and may act on gene expression and activation of signaling pathways, leading to the upregulation of genes encoding keratin-associated proteins.^10^

Most clinical studies with topical minoxidil in women have been carried out with a 2% solution applied twice daily.^11^ A meta-analysis of these studies showed a mean difference of 12.4 hairs/cm^2^ in the 2% minoxidil group when compared with the placebo after 24 weeks of treatment.^11^ The use of 5% minoxidil solution twice daily and 5% minoxidil foam used once daily showed no significant difference in response when compared to the 2% solution applied twice daily.^12^, ^13^

**Figure 1:**
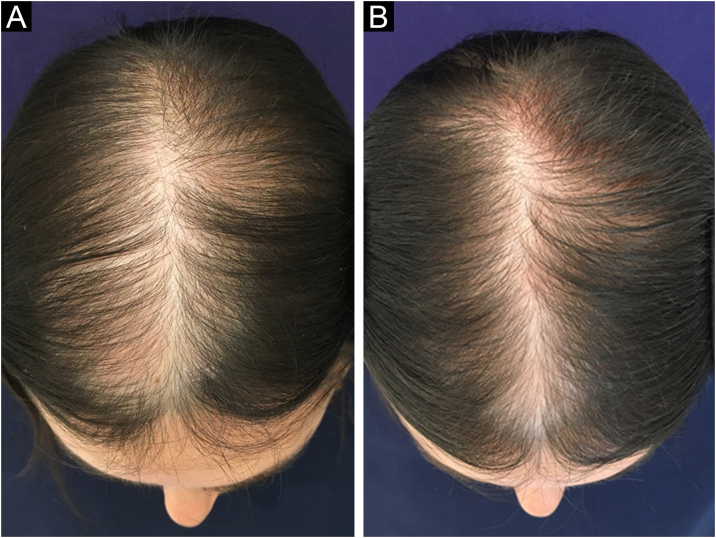
Clinical improvement in a patient with FPHL using minoxidil 5% solution 1× a day. (A) Before treatment. (B) After six months of treatment.

In Brazil, minoxidil is only marketed as a 5% solution. Due to cosmetic issues and as a matter of practical use, 5% minoxidil once daily is preferred over 2% minoxidil twice daily for the treatment of FPHL, although the 5% solution is not recognized by the regulatory organizations (FDA in the United States or ANVISA in Brazil), for use in females. In 2014, the FDA approved the use of 5% minoxidil in foam vehicles for the treatment of FPHL (Fig. 1).

Minoxidil is a prodrug. Thus, to exert its effect (both vasodilator and follicle-stimulating), the minoxidil base needs to be converted to its sulfated form by the enzyme minoxidil sulfotransferase.^7^, ^14^, ^15^ Sulfotransferase is present in several tissues and is highly expressed in the liver.^16^ It can also be found in the outer root sheath of the hair follicle.^14^ There is an interindividual variation in follicular sulfotransferase activity, and patients with higher enzyme activity have been found to show a better therapeutic response.^17^

When obtained from compounding pharmacies, minoxidil sulfate is often used instead of minoxidil base due to its easier solubilization. Apparently, this alternative could even be advantageous due to the lack of need for metabolization, but minoxidil sulfate has a high molecular weight and low skin penetration. Therefore, it is important that, when choosing a compounded product, the clinician specifies in the prescription that minoxidil base should be used.^15^

The most common adverse effects of topical minoxidil are hypertrichosis in the facial region and local reactions on the scalp, such as pruritus, burning sensation, erythema, papules, or pustules. The frequency of these manifestations depends on the concentration of minoxidil and the type of vehicle, ranging from 1.9% to 5.7% in different studies.^9^ Although minoxidil can cause contact dermatitis, the active agent that most frequently leads to this condition is propylene glycol, a substance often present in the vehicle.^18^ In these cases, symptoms will resolve with the change to propylene glycol-free topical minoxidil.

About 18% of patients using topical minoxidil experience a transient increase in hair loss in the first few weeks of treatment (shedding); this occurs due to the shortening of the telogen phase.^19^ It is important that patients be advised of this possibility to avoid early treatment discontinuation.

Adherence is essential for a successful treatment. The first results are seen after four to six months, and to maintain them, minoxidil must be continued indefinitely.^20^, ^21^ In addition to adverse effects, premature discontinuation may occur due to perceived unsatisfactory results or problems with the daily use of the topical treatment, due to changes in hair texture and difficulty to style them.

Topical minoxidil is the therapy with the highest level of evidence for the treatment of FPHL and, although it has been in use for a long time, it remains the first-line treatment.

### Oral minoxidil

Although topical minoxidil is effective for treating FPHL, 30% to 60% of patients who use it do not show improvement.^6^, ^22^ Even for patients without side effects, adherence to the topical treatment can be a problem. The use of oral minoxidil for FPHL aims to increase the potency and improve adherence to treatment due to its greater convenience when compared to topical application.

The main limitation of using oral minoxidil for the treatment of FPHL is its possible side effects. When used for the treatment of arterial hypertension, at doses of 10 to 40 mg/day, the main adverse effects are tachycardia, edema, and hypertrichosis.^7^ Considering that the effects are dose-dependent, the use of oral minoxidil at low doses minimizes adverse effects, preserving a certain stimulatory action on the hair follicle.

The first study on oral minoxidil for the treatment of FPHL was a prospective evaluation of 100 patients who used it at a dose of 0.25 mg plus spironolactone 25 mg/day, published by Sinclair in 2018.^23^ The study reported an improvement in the clinical scale and in hair loss.

An Iranian study that included 72 patients compared this same dose of 0.25 mg/day of oral minoxidil versus 2% topical minoxidil in FPHL. An improvement in hair density and hair shaft thickness was observed in both groups, and there was no difference between them.^24^

**Figure 2:**
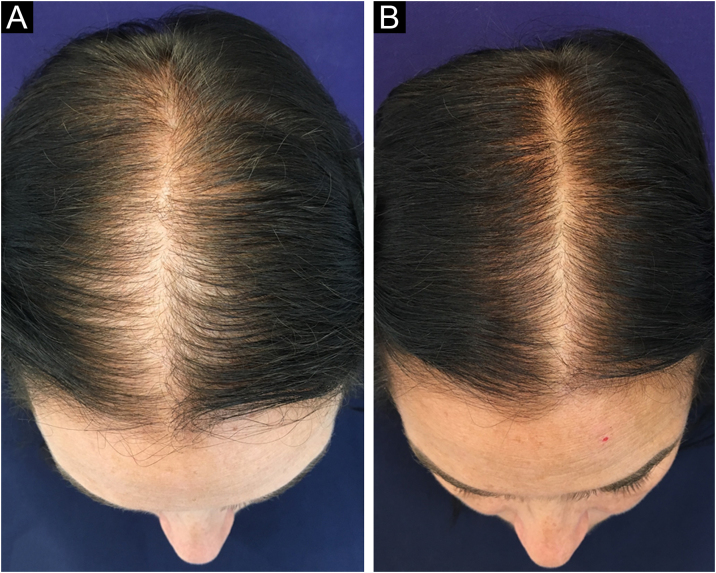
Clinical improvement of a patient with FPHL using oral minoxidil 1 mg/day. (A) Before treatment. (B) After six months of treatment.

In 2019, Ramos et al. published an open-label randomized clinical trial comparing oral minoxidil 1 mg/day vs. 5% topical minoxidil once a day in 52 Brazilian women with FPHL. Both groups showed improvement in hair density and in the photographic evaluation, with no difference between them. However, there was a trend towards superiority of the oral minoxidil group in relation to the topical group, which perhaps might have been better demonstrated in a larger sample^25^ (Fig. 2).

The clinical effect of oral minoxidil seems to correlate with its dose. In a randomized double-blind clinical trial of 30 patients with FPHL, Silva et al. found that oral minoxidil 1 mg was superior to oral minoxidil 0.25 mg daily.^26^ In the first study, patients using oral minoxidil 1 mg showed an increase in hair density of 20.1 hairs/cm^2^ and in the second study, 27 hairs/cm^2^.^25^, ^26^

There are no published series of women with FPHL using daily doses higher than 2 mg of oral minoxidil, despite anecdotal reports of individuals using up to 2.5 mg daily. There are also no data on the combination of topical and oral minoxidil for the treatment of FPHL.^27^

Recently, the sublingual route has been proposed as an alternative to the oral route to improve the bioavailability of minoxidil to the hair follicle and minimize possible adverse cardiovascular effects. ^28^ There are still no comparative studies between the two administration routes.

A recent retrospective study evaluated the adverse effects of oral minoxidil in 220 men and 215 women with AGA.^29^ Among women, the most frequent adverse effects were hypertrichosis (54%), headache (10%), lower limb edema (9%), dizziness (7%), insomnia (7%), and palpitations (4%). Forty-four percent of female patients experienced a temporary increase in hair loss at the start of the treatment. Although frequent, adverse effects were mild and well tolerated and only 13% of the patients discontinued treatment.^29^ Episodes of hypotension are infrequent with the use of oral minoxidil for AGA, as despite its potent antihypertensive effect it has little hypotensive effect in normotensive individuals.^30^

The use of oral minoxidil for treatment of AGA, both in men and women, has become increasingly frequent in recent years. This rapid increase was due to the fact that minoxidil is easily used and shows good adherence, as well as the perception of good clinical results. Despite the rapid growth in popularity, larger clinical trials comparing different doses and their outcomes versus traditional topical therapy are still necessary.

### 5α-reductase inhibitors

The action of testosterone and, mainly, of dihydrotestosterone (DHT), its metabolite produced by the action of the 5*α* -reductase (5*α*R) enzyme is crucial in the development and evolution of AGA in man.

Although the evidence showing the participation of androgens in the pathophysiology of AGA has been clearly demonstrated only in men, it has been assumed that they also play a role in women.

The efficacy of 5αR inhibitors (finasteride and dutasteride) in the treatment of male AGA corroborates the importance of DHT in its physiopathogenesis. However, finasteride 1 mg/day (standard dose for treating male AGA) has not been effective for treating FPHL in postmenopausal women.^31^

In an uncontrolled prospective study, 37 patients used finasteride 2.5 mg/day for 12 months. Twenty-three patients (62%) showed improvement in the photographic assessment and 12 (32%) showed improvement in the hair density score.^32^

Another prospective uncontrolled study evaluated finasteride at a dose of 5 mg in 87 women. After one year of treatment, 81% of patients showed improvement in the photographic assessment and there was an increase in hair density of 17 hairs/cm^2^.^33^ Randomized clinical trials evaluating doses ranging from 2.5 to 5 mg/day of finasteride in women have not yet been performed.^34^

Finasteride is a type 2 5αR inhibitor and a dose of 1 mg/day leads to a reduction of about 70% in serum DHT levels. Dutasteride inhibits 5αR type 1 and 2 and a dose of 0.5 mg/day reduces DHT levels by about 90%. Due to its greater potency, dutasteride may be an interesting alternative, but evidence supporting its use in FPHL is still scarce.^34^

The lower response of women to 5αR inhibitors probably occurs due to the involvement of non-hormonal mechanisms in the pathophysiology of FPHL, which are still not fully understood.

Finasteride 1 mg/day and dutasteride 0.5 mg/day have a package insert indicationin Brazil just for the treatment of male AGA. Its use in women is off-label and it should be used with caution in women of childbearing age due to its teratogenic potential (feminization of the male fetus). ^34^ There are no elements that clearly indicate a greater risk of breast cancer in users of 5αR inhibitors, yet caution remains necessary regarding their use in women at high risk for breast cancer.

There are no studies evaluating the performance of the combination of 5αR inhibitors and minoxidil (topical or oral) in FPHL.

### Other antiandrogen drugs

Androgen receptor antagonists are frequently used in the treatment of FPHL, especially when the patient shows signs of hyperandrogenism. Despite their routine use, there is still no high-quality evidence to support the use of these drugs, and their performance associated with minoxidil (oral or topical) remains unknown.

#### Spironolactone

This is a potassium-sparing diuretic and antagonist of aldosterone receptors and androgen nuclear receptors (NR3C4). The doses used for the treatment of FPHL range from 25 to 200 mg/day. In an open prospective study, 40 women received spironolactone 200 mg/day and 40 women received cyproterone 50 mg/day (or 100 mg for ten days a month if premenopausal) for a period of 12 months. In the photographic evaluation, 44% of the patients showed improvement with treatment and there was no difference between the groups.^35^

The most frequent side effects are fatigue, mastalgia, menstrual irregularity and hypotension. Hyperkalemia is a concern in patients taking spironolactone; however, a retrospective study of 974 healthy women taking spironolactone found no increased frequency of hyperkalemia in this population after starting the treatment.^36^ A systematic review found no association between spironolactone use and increase in breast cancer.^37^

#### Cyproterone

It inhibits the release of the gonadotropin-releasing hormone (GnRH) and is a competitive antagonist of androgen nuclear receptors (NR3C4). It is commercially available in 50 and 100 mg tablets, and it can also be found in much smaller doses (2 mg) associated with ethinyl estradiol in combined oral hormonal contraceptives (COHC).

The efficacy of cyproterone was evaluated in a randomized study that divided 66 women into two groups: 1) 2% topical minoxidil in association with COHC (gestodene 75 μg and ethinyl estradiol 30 μg); and 2) cyproterone 50 mg/day for 20 days (every 28 days) in association with COHC (cyproterone 2 mg and ethinylestradiol 35 μg). After 12 months of treatment, the minoxidil group was superior to the cyproterone group (6.2 hairs/0.36 cm^2^ increase vs. 2.4 hairs/0.36 cm2 reduction). However, cyproterone was more effective in the presence of clinical signs associated with hyperandrogenism, such as acne, menstrual irregularity, and obesity.^38^ The most frequent side effects of cyproterone are menstrual irregularity, changes in libido, mastalgia and weight gain.

#### Flutamide

This drug has a potent antiandrogenic action with package insert indication for treatment of prostate cancer by inhibiting androgen nuclear receptors (NR3C4). Due to its high potency, flutamide was frequently used to treat acne, seborrhea, and hirsutism, in addition to FPHL. However, the use of this drug for this purpose was prohibited after cases of severe acute hepatotoxicity were described.^39^

#### Bicalutamide

This is an antiandrogen, also used in the treatment of prostate cancer by inhibiting androgen nuclear receptors (NR3C4), but it has a longer half-life and lower risk of hepatotoxicity when compared to flutamide. Recently, two retrospective studies evaluated the use of bicalutamide in the treatment of FPHL.

The largest of them evaluated 316 patients who used bicalutamide in doses ranging from 10 to 50 mg a day. Two hundred and twenty patients (70%) used the 10 mg dose. One hundred and thirty-eight patients who completed six months of treatment were evaluated for efficacy. There was a 20% reduction in the Sinclair clinical scale.^40^ The second study evaluated 44 women with FPHL who used doses of 25 (15 patients) and 50 mg (29 patients) daily. Thirty-two patients underwent evaluation after six months with a 27.5% reduction in the Sinclair scale.^41^ In both studies, the efficacy results are limited by the concomitant use of other drugs for FPHL by most patients. The most frequent side effects were mild alteration of transaminase levels, mastalgia, amenorrhea, and peripheral edema.^42^

To increase treatment safety, it is suggested to perform laboratory tests (whole blood count, transaminases, alkaline phosphatase, gamma-glutamyl transferase, bilirubin, prothrombin time, creatinine, urea, sodium, potassium levels, and lipid profile) before treatment, and after 4, 12 and 24 weeks.^42^

The combination of oral minoxidil and bicalutamide (10‒25 mg/day) has been found to reduce the incidence of hypertrichosis, allowing the use of higher doses of oral minoxidil.^43^ However, this needs to be confirmed in prospective studies.^43^

None of the abovementioned antiandrogenic drugs are safe during pregnancy; therefore, it is essential to use highly effective contraceptives during the premenopausal period.

Oral hormonal contraceptives (OHC) act as hormone blockers leading to an antiandrogenic effect, in addition to increasing SHBG (sexual hormone binding globulin) levels. This effect can be maximized when the estrogenic fraction is associated with progestins with antiandrogenic action. In addition to cyproterone, other progestins such as drospirenone and chlormadinone also have this characteristic.^44^ One should take into account the contraindications for the use of OHC, such as obesity, risk of thrombophilia, breast cancer, liver disease and migraine.

#### Alpha estradiol

Alpha estradiol is a stereoisomer of the 17-beta-estradiol hormone with a low affinity for estrogen receptors. Its probable mechanism of action in the treatment of AGA would be the local inhibition of 5-alpha-reductase. The hair follicle expresses estrogen receptors, which modulate the cycle, prolonging the anagen phase. In Brazil, alpha estradiol is available for topical use in a 0.025% solution.

There is no robust evidence for the benefit of alpha estradiol in the treatment of FPHL. In a randomized comparative study for the treatment of FPHL that compared topical minoxidil and 0.025% alpha estradiol, there was no improvement in the group using the latter after six months of treatment. ^45^

The performance of topical alpha estradiol in association with minoxidil (oral or topical) is not known, nor with other antiandrogenic drugs.

### Prostaglandin analogues

Prostaglandin analogues are important modulators of follicular activity. They stimulate both keratinocyte and melanocyte activity, promoting hair growth and pigmentation.^46^, ^47^

Latanoprost, a prostaglandin F2α (PGF2α) analog, is used in the treatment of open-angle glaucoma as a 0.005% ophthalmic solution. The side effects include increased length, thickness, and pigmentation of eyelashes.^46^ In a double-blind, randomized study including 16 male patients with mild AGA, there was a significant increase in hair density (terminal and vellus hair) after eight weeks using 0.1% topical latanoprost, when compared to the baseline picture and placebo group, (an increase of 32 hairs/cm^2^ over the placebo).^47^

Bimatoprost is a synthetic prostamide, a PGF2α analogue, originally used in the treatment of ocular hypertension and open-angle glaucoma. Its daily 0.03% topical use has been approved by the FDA for patients with eyelash hypotrichosis.^46^

Despite their interesting follicular action, the use of prostaglandin analogues is still limited in the treatment of FPHL due to the lack of clinical evidence and the high cost for use in large areas of the scalp.

### Nutraceuticals

Micronutrients may play an important role in alopecia by acting on hair follicle development and immune cell function.^48^ Moreover, nutritional alterations are associated with telogen effluvium, which affects the outcome of FPHL treatment.

Several vitamin supplements and natural products such as saw palmetto, caffeine, melatonin, marine extracts, rosemary oil, procyanidin, pumpkin seed oil, and cannabidiol oil have been described in the treatment of AGA and telogen effluvium.^48^, ^49^

Lower serum levels of vitamin D and ferritin have been observed in patients with non-cicatricial alopecias compared to the healthy population.^50^, ^51^ Nevertheless, there is yet no evidence of FPHL improvement with the supplementation of these substances. There is also no evidence of the benefit of biotin and zinc supplementation in healthy patients with FPHL.^49^

Despite being very frequently used, both by spontaneous patient demand and medical advice, there are still no clinical studies demonstrating consistent evidence of the benefit of using nutraceuticals or specific supplements in the treatment of FPHL.

## Procedures

### Mesotherapy

Mesotherapy or intradermotherapy is a minimally-invasive technique that consists of infusing a mixture of active pharmaceutical agents in diluted doses intradermally.^52^ Once administered, the substances apparently achieve a more intense and lasting effect due to greater local bioavailability, in addition to potentially reducing systemic adverse effects.^53^

The active agents delivered through multiple injections directly to the affected sites comprise molecules already used through other routes of administration, such as minoxidil, finasteride, dutasteride, growth factors, panthenol, biotin, and steroids.^52^

Uzel et al. conducted a clinical trial comparing intradermal injections of sterile 0.5% minoxidil solution with injections of 0.9% saline solution. Fifty-four patients were divided into two groups and received weekly applications for ten weeks. In the treatment group, there was an increase in the ratio terminal to vellus hairs (p < 0.001). However, the increase in density in the treatment group was not statistically greater than that of the placebo group (p = 0.54).^54^

A comparative study evaluated 126 women with FPHL who were divided into two groups. The first group received injections of 0.05% dutasteride associated with biotin, panthenol, and pyridoxine and the second received saline solution. The patients received 12 injections over an interval of 18 weeks. On the photographic assessment, improvement was observed in 62.8% of the patients in the treatment group and 17.5% in the control group (p < 0.05).^55^

The weekly frequency and discomfort of the infiltration may limit the adherence of some patients to mesotherapy treatment. One alternative would be the use of quarterly dutasteride intradermal injections, due to their longer half-life, but controlled studies are still necessary to evaluate the performance of this therapeutic regimen. ^56^

The main criticisms related to mesotherapy concern the lack of standardization regarding the form of application, the active agents used, and the frequency of sessions.^52^ Pain can be a limitation of this complementary therapeutic modality. To reduce pain in patients undergoing scalp mesotherapy, non-pharmacological options such as vibration anesthesia devices were considered safe, effective, and simple to handle, providing tactile distraction at the application site and, therefore, comfort during the procedure.^57^

In addition to local pain caused by punctures and mild headaches, some complications such as persistent edema, micro bacterial infection, urticaria, skin necrosis, panniculitis, achromia, and cicatricial alopecia have already been reported. However, it is believed that part of these adverse effects is more often related to inadequate clinical indication, inadequate asepsis, use of non-sterile material, and even the performance of the procedure by a non-medical professional, than to mesotherapy itself.^53^

Special attention should be paid to the use of synthetic growth factors in the mesotherapy mixture. The literature supporting its use is very scarce, and there are reports of cases of melanoma arising after injections of growth factors as a therapy for telogen effluvium.^58^ Another reported complication was temporary frontal edema after mesotherapy for AGA. The authors have correlated edema with the use of injectable lidocaine, which is one of the formula components, and higher injected volumes are related to this side effect. All patients had a favorable outcome after one to four days with the use of cold packs alone.^59^

Mesotherapy can be considered an adjuvant option for the treatment of FPHL, although more studies are necessary to standardize the active agents, their concentrations, the periodicity of applications, and the number of sessions indicated. Although it is minimally invasive, it is essential that it be performed by a medical professional with clinical and pharmacological experience, following aseptic regulations consistent with the procedure, aiming to prevent the aforementioned complications.^60^

The performance of injectable procedures in association with minoxidil (oral or topical) is not known, nor with other antiandrogenic drugs in the treatment of FPHL. Finally, in addition to the fact that the active agents are not approved by the FDA or ANVISA for this route of administration, they contain products with questionable sterilization.

### Microneedling

Microneedling is a minimally-invasive technique that consists in using sterile microneedles for repetitive skin punctures that create micro-channels in the skin.^52^, ^61^ The proposed action mechanism to increase hair growth is due to the release of platelet growth factors (TGF-alpha, TGF-beta, platelet-derived and vascular endothelium-derived growth factor), which would subsequently result in neovascularization, increased collagen, and elastin production, and stimulation of the expression of genes related to hair growth.^52^, ^61^

There are several devices that can be used to perform the procedure. The most traditional are rollers, stamps, and electric pens, containing needles that vary in number and length.^61^

Microneedling is a low-cost, quick-to-perform procedure with a simple learning curve. It can be done under topical anesthesia or anesthetic block for greater patient comfort.^52^, ^62^, ^63^

After anesthesia, the area to be treated must be washed with saline solution and ethanol to ensure an aseptic field. The pen has adjustable penetration speed and depth and must be moved linearly, lifting the device after each application, in a total of up to three applications per location.

In the case of the needled roller, various applications in several directions can be made until blood droplets appear over an area of homogeneous erythema on the scalp, although the ideal endpoint for this procedure remains controversial.^62^

Although some studies point to favorable results of microneedling in the treatment of AGA, the evidence for the benefit of this treatment is still of poor quality.^61^ In an open study, Starace et al. evaluated 29 patients with FPHL, who showed an increased density of 19.57 hairs per cm^2^ in the frontal area after three microneedling sessions four weeks apart.^63^ There have been reports of tolerable pain and transient lymphadenopathy.

Microneedling can be an adjuvant option for the treatment of refractory FPHL or for those who do not wish to undergo the standard clinical treatment. Its real effectiveness, as well as the ideal frequency of sessions and length of needles, must be established by studies with specific designs. There is some controversy as to whether the puncture should be deep enough to penetrate the barrier and deliver the medication or whether it should be superficial enough to produce minimal damage and little discomfort during the procedure. Needles of 0.5 to 2.5 mm have already been studied, with favorable results.^61^, ^64^

As an adjunctive treatment for FPHL, microneedling is a relatively new procedure and lacks standardization about the best devices, frequency of applications, endpoint, and the number of sessions.^62^ Likewise, it is not known whether the fibrosis resulting from several sessions of microneedling can interfere with the technical difficulty of performing follicular transplants later. The topical use of sterile soluble pharmaceutical actives after microneedling with drug delivery intention has not yet been systematically studied regarding additional efficacy, nor are these actives authorized by regulatory organizations (FDA and ANVISA) for use through this route of administration.

### Microinfusion of drugs into the skin(MMP®)

The MMP® technique aims to promote drug infusion (drug delivery) and stimulation with needles in the epidermis and superficial dermis. For that purpose, it uses a tattoo machine and appropriate needles, which meet the adequate principles of equipment sterilization and disposal of needles after the procedure, which can be done in an outpatient setting.^65^ Mixtures with the infused actives are sterile and can be the same as described in the mesotherapy section.

Even when the drug infusion function is not used, at the physician discretion, the punctures produced by the machine can already induce an effect similar to that of using rollers, depending on the depth achieved.^65^

One study demonstrated that medication delivery through the MMP® technique occurs uniformly in the superficial dermis, requiring only a small volume of actives, in addition to not creating a “mass” effect, which reduces the chance of local adverse reactions. Moreover, the fact that the operator can adjust drug delivery and infusion depth also increase the safety of this technique.^66^

Pain, which is an undesirable effect of the microneedling technique, can also occur with MMP®.^65^ The folding technique consists of folding the skin of the area to be treated, between the thumb and index finger of the non-dominant hand of the physician performing the procedure. It may be useful in reducing pain during the procedure, as a result of changing the perception of pain by tactile stimulation and distancing the needles from the galea, which is richly innervated.^67^ Other more serious side effects have not been reported to date.^65^

Although apparently promising as adjuvant therapy, the literature on the subject is still scarce, especially with regard to FPHL. Moreover, some important recommendations remain empirical, such as the precise indications for the technique, the number of sessions necessary for the perception of the initial results, the ideal interval between sessions, and the need to recommend maintenance applications. The pharmaceutical actives used still lack authorization from the regulatory organizations (FDA and ANVISA) for use through this route of administration.

### Platelet-rich plasma (PRP)

PRP is a technique that consists of the intradermal injection into the scalp of a concentrated autologous platelet preparation, which releases several growth factors, such as platelet-derived growth factor (PDGF), transforming growth factor beta (TGF-β), vascular endothelial growth factor (VEGF), Epidermal Growth Factor (EGF), and insulin-like growth factor-1 (IGF-1). All of these growth factors play an important role in the hair cycle, as they stimulate stem cells located in the bulge, responsible for the follicular unit growth.^68^

A randomized controlled clinical trial in women with FPHL evaluated the effect of two monthly sessions of PRP against a placebo. This study found that, after 24 months of follow-up, there was an improvement in the photographic assessment in 57% of patients with PRP, versus 7% of improvement in patients receiving saline solution.^69^

Recently, a meta-analysis including 15 clinical trials evaluating PRP for AGA described an increase in hair density of 36.8 hairs cm^2^ (95% CI 22.6–51.1). However, many of these studies are limited by the small sample size and lack of adequate blinding.^70^

PRP is a promising adjuvant treatment in the management of FPHL; however, there is yet no standardization regarding blood preparation, centrifugation regimens, platelet density, need for platelet activation, and the technique of application.^70^, ^71^ In a recent review, Stevens J. et al. suggested the following protocol: single centrifugation with platelet enrichment of three to six times the mean concentration of whole blood, proposed session interval of three monthly sessions, followed by quarterly sessions in a year.^68^

Both PRP and microneedling promote the local release of growth factors and epidermal and upper dermal trauma, but there are no comparative studies between the two treatment methods.

Up to now, the use of PRP for dermatological treatments in Brazil is still vetoed by the Federal Council of Medicine.

### Botulinum toxin

Oxidative stress, perifollicular microinflammation, and microvascular insufficiency have been reported as factors associated with AGA.^72^, ^73^ Goldman et al. observed that in AGA, bald areas have lower oxygen (O_2_) levels than non-bald ones, suggesting some degree of microvascular insufficiency.^74^ Moreover, the conversion of testosterone into DHT preferentially occurs in an O_2_-poor medium, and therefore, an increased blood supply would reduce local hypoxia and thus could be beneficial in the treatment of AGA.^75^, ^76^

It is known that DHT induces the production of transforming growth factor-β1 (TGF-β1) in the dermal papilla cells, having an important action in follicular epithelial cell growth suppression. Thus, TGF-β1 is a pro-apoptotic factor, with a relevant role in the onset of AGA, and antagonizing it could constitute a way to prevent disease progression.^77^

Botulinum toxin (BT) is a neurotoxin obtained from the bacterium *Clostridium botulinum*, which inhibits muscle contraction by blocking acetylcholine. The reasoning for the injectable use of BT in AGA would be to promote relaxation of scalp muscles, reducing muscle pressure on the perforating arteries and potentially increasing blood and O_2_ flow to the bald areas. Therefore, there would be a reduction in tissue DHT by the effect known as “washout” and, consequently, a lower rate of follicular miniaturization considered the main pathophysiological basis of the disease.^75^ As an additional mechanism, recently, Shon et al. suggested that intradermal injection of BT may be a possible treatment option for AGA, as it inhibits TGF-β1 secretion from hair follicles, thus contributing to the aforementioned effects.^77^

A prospective study evaluated 37 men using finasteride and minoxidil for AGA, who underwent application of botulinum toxin in half of the scalp, and the other half was injected with saline solution. After six months, there was a greater increase in hair density, 47.43 (±25.72) in the area treated with botulinum toxin compared to the control side, 21.68 (±11.09; p < 0.05).^78^ Despite the suggestion of applying the intradermal units 1 cm apart from each other, there is still no consensus on how to apply it and the interval regimen. The proposal to use botulinum toxin for the treatment of AGA is recent, promising, and has a good safety profile. However, systematic studies are required to prove its effectiveness in both men and women.^76^

### Photobiomodulation

Photobiomodulation or low-level laser therapy (LLLT) is a treatment modality used for patients with alopecia approved by the FDA in 2009.^79^

Photobiomodulation therapy devices typically contain laser diodes or light emitting diodes (LEDs) that emit light continuously (continuous wave) or in short, rapid pulses (pulsed emission). While lasers produce coherent, monochromatic light with a more focused radiation area, LEDs produce incoherent light with a broader emission spectrum and greater radiation field. Coherent laser light has historically been considered more effective than LED light, but it remains controversial whether one light source actually offers a clinical benefit over the other.^80^

The exact mechanism of action of photobiomodulation in the treatment of AGA is still unknown. It has been suggested that LLLT promotes keratinocyte and fibroblast mitosis through the stimulation of anti-inflammatory and antioxidant cytokines, in addition to a perifollicular vasodilator effect.^79^ Studies in both sexes showed that LLLT would be a well-tolerated therapy with no reported adverse effects. From an effectiveness point-of-view, most of the time, the results in AGA point to a quantitative increase in hair density and reduction in hair loss when compared to the placebo group.^79^

There are several devices for home use in the form of caps, helmets, headbands, and combs. Each one has a different amount of laser points and some are associated with LEDs, making it inappropriate to compare results between one device and the other.

The number of weekly applications and the duration of each application varies according to the device. Clinical response, according to studies, begin around four months after starting treatment.^79^

Regarding the energy provided by the devices, review studies have shown variations between 630 and 808 nanometers. From the biomolecular point of view, LLLT showed an increase in the main extracellular matrix proteins, which corroborates the clinical perception of increased hair diameter in users with AGA. Interestingly, the low-frequency (less than 60 minutes a week) treatment regimen seems to be more effective than the high-frequency one (more than 60 minutes a week).^81^

LLLT is an option as an adjuvant treatment for FPHL or as an alternative for those who do not wish to undergo clinical or surgical treatments. There is no evidence to support the use of LLLT sporadically prior to minimally invasive hair procedures, such as mesotherapy, microneedling, and MMP®.^81^

## Hair restoration surgery (hair transplant)

Despite the several treatment options, many patients do not achieve the desired result, either because of poor response to therapy or because of an initially advanced clinical condition. In these cases, surgical restoration may increase hair volume in a given area by distributing follicular units, obtained from another region of the patient scalp.^82^

Hair restoration surgery in FPHL is an exceptional indication and must be performed under very specific conditions. It should not be considered an isolated procedure, much less promoted as a cure for FPHL. Maintaining clinical treatment is essential to prevent the expansion of hair loss to other areas.

In the initial consultation of the patient with FPHL, it is up to the hair restoration surgeon to obtain a thorough clinical history and perform accurate examination of the entire scalp. In addition to the areas most affected by FPHL, the evaluation of the donor area is essential to verify the number of follicular units (FUs) that can be removed. It is important to exclude other types of alopecia that are not amenable to surgical treatment, such as alopecia areata, chronic telogen effluvium, and active cicatricial alopecia.

It is necessary to listen to the patients complaints and understand their expectations. Often, the patient expresses the desire to cover a larger area than is possible; so one should advise them that the anterior part of the scalp (frontal and central) should be filled as a priority, as they are the most visible regions. If the anterior line is affected, it must be created irregularly and with the most delicate FUs, so as not to show that it is an artificially constructed line. Frontotemporal recess areas are also a frequent complaint and the specialist should draw a line consistent with the female outline.

The ultimate goal of hair restoration in FPA patients is to achieve good density in the recipient area. For this, either you have a large reserve of FUs or you must limit the area to be transplanted. In most cases, when examining the donor area, it can be observed that there is no great density or the hairs are equally thin when compared to the regions affected by miniaturization. In this situation, surgical treatment should not be proposed. These are cases with a good indication when there is a large number of follicles and thicker hairs in the posterior region of the scalp, than in the area to be covered.

When well indicated, the specialist can assure the patient that the result of the procedure will, in most cases, be natural and show good density. The FUs will be transplanted individually, inside minimal orifices, thus not creating visible scars and without the appearance of tufts that were popularly called “doll hair” in the past. There are two approaches to be discussed with the patient regarding the removal of FUs from the donor area and these techniques are commonly referred to by their acronyms.

**Figure 3:**
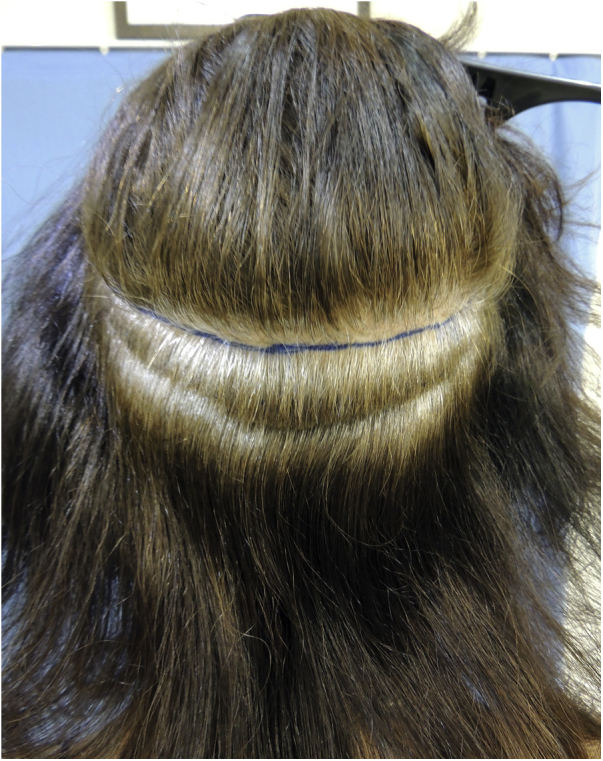
Area from which the skin strip will be removed for the extraction of follicular units (FUT - Follicular Unit Transplantation technique).

Follicular unit transplantation (FUT) refers to the removal of a skin strip from the scalp and, consequently, it will leave a single horizontal scar in the donor area (Fig. 3). This skin strip will be submitted to microscopic dissection by the team to produce the FUs. It is important to assess the elasticity of the scalp, as the stip width should not exceed the possibility of approximation of the edges without excessive tension.

**Figure 4:**
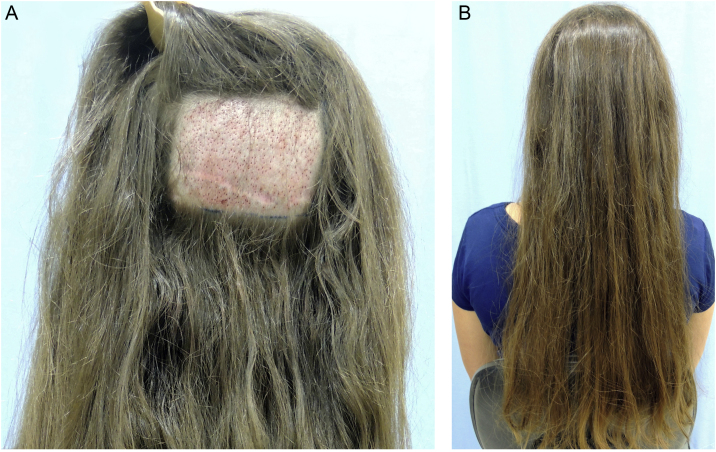
(A) Shaved area for removal of follicular units using the FUE (Follicular unit extraction) technique. (B) Long hair covering the shaved area.

Follicular unit excision (FUE) allows the excision of FUs directly from the scalp, using specific equipment: a 0.85‒1.00 mm micro-punch that can be coupled to the rotation machine or it can be used manually. The consequence of this approach is thousands of micro-scars homogeneously spread throughout the donor area (Fig. 4). In both techniques, the insertion of FUs into the receiving areas will be performed in the same way, either using delicate tweezers or implanters.

The FUT technique does not require shaving the hair, which is usually advantageous for women as they often do not want to have their heads shaved and are not afraid of a linear scar. Usually, the FUE technique is performed by shaving the hairs in the donor area but shaved “windows” can be made by keeping the long hair above these sectors to cover the donor area. It is possible to remove long hairs using the FUE technique, but with much greater technical difficulty and increased surgical time. However, both are well indicated, depending on the patient choice and the specialist technical knowledge.

During the appointment, in addition to deciding on the best technique for the case, the patient should also be told how many sessions will be planned. Usually, the performance of only one step is intended, but a second session may be necessary, at least one year apart. The patient should also be submitted to preoperative exams, as well as surgical risk assessment.

A second appointment is scheduled, called a preoperative consultation, which is used to check the exams, answer questions, review the planning and scheduling, take preoperative photos, sign the consent forms, and receive instructions for the day of the surgical procedure. The procedure can be performed with a sterile technique, in an outpatient operating room, which allows constant monitoring of the patient. Local anesthesia is employed, with anesthetic blockade of the scalp. Intravenous sedation performed by the anesthetist is not mandatory, but it brings comfort to the patient as it allows painless infiltration and decreases the perception of the surgical time, which usually lasts four to six hours.

Daily activities can be resumed on the following day, but it is prudent to rest for four to six days, due to possible discomfort in the donor area and a slight swelling in the frontal area. The incision is not visible in FUT and the stitches will be removed after eight to postoperative days. When the FUE approach is used, one can leave the hair long enough to cover the shaved area and no sutures are used in this technique (Fig. 4). After two weeks, the patient can resume sports activities, sea bathing, etc.

It is important to inform about a possible telogen effluvium that may affect the hairs in the transplanted area. This phenomenon occurs from the first month after the procedure, and – if intense – it may be necessary to use some concealer (e.g., scarf, hat) in social situations. The transplanted follicles begin their growth after three to six months postoperatively. The final result can be observed after one year when pictures are taken and compared with the pictures taken before surgery.

Indicating hair restoration surgery in patients with FPHL requires technical knowledge, as well as an accurate capacity to estimate whether there will be an appreciable gain in hair volume in the rarefaction area. If the patient expectation is unrealistic, especially in those with body dysmorphic disorders, or when the donor area is not in good condition, it is preferable not to indicate surgery.^83^

## Camouflage methods

In the FPHL therapeutic arsenal, hair loss camouflage methods represent much more than a simple cosmetic tool. They can minimize psychological damage, improve self-esteem, social adjustment, and quality of life of affected patients, especially when there is no therapeutic response, as well as in more advanced cases.

### Hair prostheses

Hair prosthesis is a more specific term than wig to describe the hair piece used in medical conditions, such as advanced stages of FPHL.

Although the medical literature on this subject is still limited, it is crucial for the dermatologist to know the peculiarities of hair prostheses, such as the types of fibers from which they can be made, the characteristics of the bases, their main fixing methods and extension of the prostheses, to better indicate them.^84^

#### Types of hair fibers

As for the type of hair fibers, prostheses can be divided into synthetic or made from natural hair. Synthetic fibers are more durable, have more affordable prices, and require simpler maintenance. The main disadvantages of this type of prosthesis are its unnatural appearance, and the impossibility of styling and exposing it to high temperatures, such as the heat of hair dryers, for instance. Prostheses made from human hair fibers provide a more natural appearance due to the gradual variation in fiber color and diameter. They behave like natural hair and therefore can be styled, colored, and even resist high temperatures. Some disadvantages of human hair prostheses include a higher cost, greater susceptibility to fading caused by sunlight and environmental damage, and they require constant maintenance.

#### Types of base or foundation

The base of the prosthesis is the place where the hair is fixed when applied to the patient. It can be shaped to cover the patient entire head or just an alopecic area. It can be made of different materials and usually tries to simulate the scalp. The main types of foundation are the “*skin*”, made with durable materials, which provides a natural look; the “*net*”, made of a delicate mesh with synthetic or human hair tied by hand that can naturally camouflage with the scalp and the “*lace*”, made of a transparent and thin nylon material.

#### Fixing methods

Some prostheses have Velcro strips or adjustable bands around their perimeter to secure them to the scalp. The most modern ones have adhesion throughout the base, allowing more stable fixing, which is important for patient confidence.

If the patient requires a prosthesis that needs to be temporarily removed, the use of clips, hairpins, or “tic-tac clips” would be adequate. The main advantage of this method is that patients can maintain their clinical treatment in parallel. The disadvantage is possible traction alopecia or hair breakage due to the clips.

Adhesive tapes are recommended for those who need adhesion for longer periods, including during sleep, bathing, sexual intercourse, or sports practice. In addition to traction alopecia and hair breakage, a disadvantage of using adhesive tape would be the possibility of it causing contact dermatitis, especially with adhesive tapes containing cyanoacrylate.

The most expensive types of prosthesis are the customized, base-vacuum ones, for which a personalized plaster cast of the individual scalp is created first. The mold is used to create a silicone or polyurethane vacuum base, a process that can be time-consuming. This prosthesis fits the scalp perfectly and does not require any other form of adhesive for fixing.

#### Extension of prostheses

Total prostheses known as “full cap” are custom-made, made from human hair, and cover the entire scalp. The partial ones vary according to the patient needs and the extension of the FPHL. They are primarily designed to cover localized hair loss areas.

The hair integration systems (interlace) integrate the prosthesis with the patient hair and constitute an option to add more density to the bald area. Generally speaking, the fibers can be natural or synthetic, the bases can cover the entire scalp or part of it, and the prostheses can be removable or fixed. Each type has advantages and disadvantages in terms of cost, natural appearance, and practicality. Hair prostheses result in an immediate cosmetic result and should be considered complementary options to clinical treatment, especially in more advanced cases of FPHL.^84^

### Sprays, powders and fibers

Sprays, powders, or fibers are methods of hair loss concealing that act as a temporary camouflage to cover areas with less density or absence of hair on the scalp.

Hair fibers, for example, consist of keratin that electrostatically adheres to the hair, giving it an appearance of more volume and, thus, disguising the areas of alopecia. Sprays, on the other hand, are more suitable for covering white hair or attenuating differences in color between hairless areas and those with hair.

The covering effect is natural and obtained immediately after the application. It does not cause any harm to the patient and is compatible with other dermatological treatments. In general, they are resistant to sweat, rain and wind, and can be removed after washing with shampoos. Because they do not have toxicity and are not absorbed, pregnant women and children can use them. Covering with these products promotes a natural and immediate result and should be considered complementary options to clinical treatment.

### Trichomicropigmentation

Trichomicropigmentation, also known as hair micropigmentation, is a non-surgical aesthetic procedure that uses conventional tattooing on the scalp and areas with hair, with the purpose of disguising unsightly scars, deformities, hair loss areas, and other conditions that cause hair thinning.^85^

The technique includes inserting microdroplets of pigment through the epidermis into the upper dermis (approximately 1.5 mm deep) using a standard tattoo instrument, which holds between one to six needles and cycles between 100 to 150 cycles per second. A tattoo is performed using a dotted pattern imitating the follicular ostia to decrease the contrast between the hair and the skin color. Thus, it produces an effect of increased hair density.^85^ The size and spacing of the dots will vary according to the professional artistic judgment.

Pigments used in micropigmentation are mainly inert, non-toxic, non-allergenic, stable in tissue and injected with approximately 6-µm gauge needles. The pigment substances differ depending on the required color. Cinnabar and mercuric sulfate are used for red coloring, iron oxide for black and brown, and cadmium sulfide is used for yellow coloring.^60^ There may be a bluish or greenish color resulting from direct exposure to ultraviolet light on the pigment through the skin. Moreover, prolonged exposure to the sun can accelerate changes in pigment hue. ^86^

This procedure must be performed by trained professionals. It has risks, such as bacterial infection and allergic reactions, and it can complicate with bleeding followed by loss of pigment when injected too superficially.^85^

**Figure 5:**
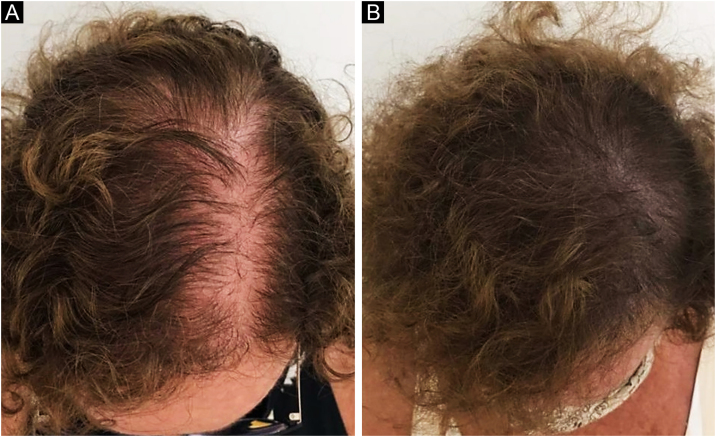
Hair loss camouflage with trichomicropigmentation.

Trichomicropigmentation is an interesting option for patients with FPHL in more advanced stages, with unsatisfactory response to clinical treatment, or with limitations to perform hair restoration surgery. Additionally, it promotes a permanent camouflage of thinning hair areas, promptly restoring self-esteem to affected patients (Fig. 5).

## Final considerations

The treatment of FPHL is a routine challenge in the practice of dermatologists due to its high prevalence, great impact on quality of life, and therapeutic options with a limited level of evidence, which often do not meet the patient expectations.

Because it is a chronic and progressive disease, treatment must be maintained for an indefinite period; however, considering that most clinical trials last up to one year, data on long-term results are scarce.

In general, milder cases of FPHL are more responsive to therapy than more extensive ones. Routine clinical and trichoscopic examination in the dermatological consultation allows the diagnosis of early cases, especially in those at higher risk, such as the ones with hyperandrogenism, users of androgenic drugs (e.g., steroids for sports performance or anastrozole), and those with a family history of AGA.

None of the drugs used for FPHL has an adequate safety profile for use during the gestational period, especially antiandrogens and 5-alpha-reductase inhibitors.

The dermatologist must be aware that adherence can be a problem and must be alert to all elements that may impact it. Adequate follow-up, with standardized clinical and dermoscopic photos, is important not only for evaluating the efficacy but also for motivating the patient to maintain the treatment.

Conducting randomized clinical trials comparing the different available treatments is essential for a better understanding of the efficacy of the available therapeutic options, as well as their associations. Moreover, it is essential to increase the understanding of the pathophysiology of FPHL, which goes beyond genetic predisposition and androgen action. This knowledge will lead to the identification of therapeutic targets, which will allow the development of more specific and effective treatments.

## Financial support

None declared.

## Authors' contributions

Paulo Müller Ramos: Approval of the final version of the manuscript; planning; drafting and editing of the manuscript; critical review of the literature; critical review of the manuscript.

Daniel Fernandes Melo: Approval of the final version of the manuscript; planning; drafting and editing of the manuscript; critical review of the literature; critical review of the manuscript.

Henrique Radwanski: Approval of the final version of the manuscript; planning; drafting and editing of the manuscript; critical review of the literature; critical review of the manuscript.

Rita Fernanda Cortez de Almeida: Approval of the final version of the manuscript; planning; drafting and editing of the manuscript; critical review of the literature; critical review of the manuscript.

Hélio Amante Miot: Approval of the final version of the manuscript; planning; drafting and editing of the manuscript; critical review of the literature; critical review of the manuscript.

## Conflicts of interest

None declared.
